# Huangqi decoction inhibits apoptosis and fibrosis, but promotes Kupffer cell activation in dimethylnitrosamine-induced rat liver fibrosis

**DOI:** 10.1186/1472-6882-12-51

**Published:** 2012-04-24

**Authors:** Cheng Liu, Gaoqiang Wang, Gaofeng Chen, Yongping Mu, Lijun Zhang, Xudong Hu, Mingyu Sun, Chenghai Liu, Ping Liu

**Affiliations:** 1 Institute of Liver Diseases, Shuguang Hospital, Shanghai University of Traditional Chinese Medicine, Shanghai, 201203, China; 2 Department of Traditional Chinese Medicine, Shanghai Public Clinical Health Center, Shanghai, 201508, China; 3 Department of Integrated Traditional Chinese and Western Medicine, Eastern Hepatobiliary Surgery Hospital, Shanghai, 200438, China; 4 Shanghai University of Traditional Chinese Medicine, 1200 Cailun Road, Shanghai, 201203, China

## Abstract

**Background:**

Previously, Huangqi decoction (HQD) has been found to have a potential therapeutic effect on DMN-induced liver cirrhosis. Here, the mechanisms of HQD action against liver fibrosis were investigated in relation to hepatocyte apoptosis and hepatic inflammation regulation.

**Methods:**

Liver fibrosis was induced by DMN administration for 2 or 4 weeks. Hepatocyte apoptosis and of Kupffer cells (KC) and hepatic stellate cells (HSC) interaction were investigated using confocal microscopy. The principle cytokines, fibrogenic proteins and apoptotic factors were investigated using western blot analysis.

**Results:**

Compared with the DMN-water group, HQD showed decreased hepatocyte apoptosis and reduced expression of apoptotic effectors, cleaved-caspase-3, and fibrotic factors, such as smooth muscle α-actin (α-SMA), transforming growth factor beta-1 (TGF-β1). However, the KC marker CD68 increased significantly in DMN-HQD liver. Confocal microscopy demonstrated widespread adhesion of KCs to HSCs in DMN-water and DMN-HQD rats liver.

**Conclusions:**

HQD exhibited positive protective effects against liver fibrosis; its mechanism of action was associated with protection from hepatocyte apoptosis and the promotion of CD68 expression in the devolopment of liver fibrosis to cirrhosis development.

## Background

Fibrosis is a wound-healing response that engages a range of cell types and mediators to encapsulate an injury. During the fibrogenesis development, many pathological factors, such as inflammation derived from Kupffer cells (KCs), angiogenesis, and hepatic stellate cell (HSC) activation, interact with each other, leading to collagen deposition [1]. Cirrhosis, the most advanced stage of fibrosis, includes inflammation as a pathological factor but this aspect is remarkably reduced in fibrosis, with septa and nodule formation being the most notable features [2]. Understanding of the pathological factor differences between fibrosis and cirrhosis may lead to the development of agents suitable for cirrhosis but not fibrosis. All these concepts derive from the striking progress in the understanding of the biochemistry and cell biology that underlies fibrosis and cirrhosis as a comprehensive pathological process involving not just a single cell type [3].

In the past 20 years, HSCs have emerged as a well-characterized cell type with a central role hepatic fibrosis hepatic fibrosis [4,5]. Recent research has shown that the microenvironment plays a key role in regulating HSC activation [6]. KCs or resident hepatic macrophages carry out an important role in modulating inflammation in liver fibrosis development [7-9]. In the case of liver fibrosis, it has been suggested that KCs produce a variety of proinflammatory cytokines, such as tumor necrosis factor (TNF)-α, interleukin (IL)-1β, and macrophage inflammatory protein (MIP)-1, which provoke HSC activation and subsequently contribute to hepatic injury. According to the published data, inflammation may be a bridge between liver injury and fibrosis that occupies for a pivotal position in fibrosis development [10].

In spite of the high incidence of hepatic fibrosis worldwide, no generally accepted antifibrogenic therapy is available. Chinese herbal medicine has been widely used for treating chronic liver hepatitis and liver cirrhosis for thousands of years. and these treatments appear to improve clinical symptoms, liver function and patient quality of life [11,12]. Huangqi decoction (HQD) is a classical recipe for treating liver injury that has a long history in traditional Chinese medicine. HQD consists of two medicinal herbs, *Radix Astragali* and *Radix et Rhizoma Glycyrrhizae*, mixed in a 6/1 (wt/wt) ratio. It has been previously reported from this laboratory that HQD exerts significant therapeutic effects in the treatment of liver cirrhosis induced by dimethylnitrosamine (DMN) in rats [13]. However, in another study, HQD has been shown not to significantly improve liver function in the process leading from fibrosis to cirrhosis [14].

To evaluate the factors critical for DMN-induced liver injury, the present study focused on the HQD’ effects on hepatocyte apoptosis, fibrosis, and inflammation regulation in the development of liver fibrosis to cirrhosis following DMN-induced liver injury.

## Methods

### Materials

DMN and Sirius red were purchased from Sigma-Aldrich (Saint Louis, MO, USA). ApopTag Fluorescein in Situ Apoptosis Detection kit (S7110) was purchased from Chemicon International Inc. (Temecula, CA, USA). For primary antibodies used in immunostaining and western blot, anti-caspase-3 rabbit antibody was purchased from Cell Signaling Technology, Inc. (Danvers, MA, USA), used at 1:1000 dilution; anti-CD68 mouse antibody purchased from AbD Serotec (Kidlington, UK), used in 1:100 dilution; anti-TNF-a and anti-IL-1β rabbit antibodies from Chemicon Internat. Inc., diluted to 0.2 μg/ml; anti-MIP-1 rabbit antibody from BioVision, Inc. (Milpitas, CA, USA), used to 0.2 μg/ml; anti-a-SMA mouse antibody from Sigma, used at 1:400 dilution; and anti-TIMP-1 and TIMP-2 from Lab Vision, Thermo Scientific, Kalamazoo, MI, USA), diluted to 2 μg/ml. Anti-TGF-β1 was obtained from R&D Systems, Inc. (Minneapolis, MN, USA), used at 1:1000 dilution,anti-HGF rabbit antibody from Enzo Life Sciences, Inc. (Farmingdale, NY, USA), used in 5 μg/ml dilution; anti-glyceraldehyde 3-phosphate dehydrogenase (anti-GADPH) mouse antibody from Kangchen Bio-tech Inc. (Shanghai, CN) diluted by 1:5000; the secondary fluorescence-labeling goat anti-mouse FITC, Cy3 antibody from Jackson ImmunoResearch Laboratories, Inc. (West Grove, PA, USA), used at 1:1000 dilution; and labeled goat anti-mouse isotype-specific antibody Alexa Fluor 647 immunoglobulin G (IgG)2a from Molecular Probes (Life Technologies, Grand Island, NY, USA), used at 5 mg/ml.

### DMN model

All procedures using animals were carried out in accordance with the guidelines presented in the “Principles for the Care and Use of Animals in the Field of Physiological Sciences,” published by the Physiological Society of China. The study protocol was approved by an ethics committee of Shanghai University of Traditional Chinese Medicine’s Animal Ethics Committee (Shanghai). Forty male Wistar rats (180-200 g) were housed in an air-conditioned room at 25°C with a 12 h light/dark cycle, were randomized into two groups, a control (n = 10) and a DMN-treated group (n = 30). DMN was administered at 10 mg/kg intraperitoneally to rats for 3 consecutive days each week for 4 weeks [15]; control rats received equal quantities of physiological saline. At the end of the second week, 3 and 6 rats from the control and DMN-treated groups, respectively, were sacrificed for fibrosis development assessment. The remaining DMN rats were further randomized into 2 groups, a DMN-water (n = 12) and a DMN-HQD group (n = 12). With continued weekly DMN treatment, the rats received a daily administration of water or HQD given intragastrically at 1 ml/100 g. At the end of the fourth week, all animals were sacrificed and liver samples collected for subsequent investigations.

### Preparation of HQD

HQD consists of crude slices from *Radix Astragali* and *Radix et Rhizoma Glycyrrhizae* mixed in a 6/1 ratio (wt/wt)*.* The herbal medicine was accredited by pharmacognosist and prepared by Shuguang Hospital. Specifically, the medicinal herbs mixture was extracted in boiling water and the resulting aqueous extracts dry-sprayed to obtain a powder and then stored at −20°C. The extract powder was weighed and used for experiments by dissolution in pure water at the desired concentrations.

### Histological analysis

Liver specimens were preserved in 4% paraformaldehyde, dehydrated in a graded alcohol series, embedded in paraffin blocks, sectioned to 5 μm-thick slices, placed on glass slides, and stained with Sirius red. Fibrosis was graded according to the method by Scheuer as follows: grade 0, normal liver; grade 1, increased collagen without formation of septa (small satellite expansion of portal fields); grade 2, formation of incomplete non-interconnecting septa, from portal tract to central vein; grade 3, complete but thin interconnecting septa, which divide the parenchyma into separate fragments; and grade 4, complete cirrhosis, similar to grade 3 but with thicker septa [16]. Three pathologists blind to the rats' treatment assignments performed pathological examinations. Fibrosis scores were given after thorough examination of three different areas of the tissue slide from each rat.

### Hepatic hydroxyproline content

Liver tissue (100 mg) was prepared for hydroxyproline (Hyp) determination using to a modified version of a method developed by Jamall [17]. Hyp liver content served as an indirect measure of tissue collagen content, expressed as μg/g wet weight (μg/g).

### Western blot analysis

Liver samples were prepared in radio immunoprecipitation lysis buffer containing protease inhibitors (Mini Protease Inhibitor Cocktail cOmplete, Roche Applied Science, Tokyo, JP) and phenylmethylsulfonyl fluoride (Ameresco Inc., Solon, OH, USA). After protein quantification, equal amounts of protein (50 μg/lane) were separated by 10 or 15% polyacrylamide gel electrophoresis (PAGE) and transferred to Immobilon-P transfer membranes (Millipore, Billerica, MA, USA), which were then blocked and exposed to antibodies. The antigens were visualized using an ECL kit for 1 min followed by exposure to Kodak film.

### Immunohistochemical staining

After deparaffinization and dehydration, microwave antigen retrieval was performed for 5 min prior to peroxidase quenching with 3% H_2_O_2_ in phosphate buffered saline (PBS) for 15 min. Subsequently, sections were preblocked with 5% bovine serum albumin (BSA) for 30 min and incubated with a primary antibody (anti-HGF, diluted to 5 μg/ml in PBS) overnight at 4°C. A negative control was treated as the other samples except that primary antibodies were replaced with PBS and no staining took place. After washing in PBS, sections were incubated with biotinylated secondary antibody for 30 min and then stained with 3,3’-diaminobenzidine (Vector Laboratories, Inc., Burlingame, CA, USA) for 2–5 min. Slides were finally counterstained with hematoxylin for 2–3 min, mounted, and examined.

### Gelatinase activity assay

Liver proteins (50 μg/lane) were electrophoresed in 10% sodium dodecyl sulfate (SDS)-PAGE containing 0.1% gelatin (Sigma-Aldrich), the SDS removed by soaking the gels in buffer containing 50 mM Tris (pH 7.6), 10 mM CaCl_2_, and 2.5% Triton X-100 (Sinopharm Chemical Reagent Co., Ltd, Shanghai, CN) for 20 min, followed by the same buffer containing 1% Triton X-100. Digestion was allowed to occur at 37°C for 24 h and gels then stained with Coomassie brilliant blue and destained until clear bands became evident.

### Immunofluorescence staining

Liver samples were excised, and immersed immediately in cryomatrix (Tissue-Tek OCT, Sakura Finetek USA, Inc., Torrance, CA, USA), and flash-frozen in liquid nitrogen; sectioned to 5 μm slices, and mounted on slides. Slides were then incubated in 5% BSA for 30 min and followed by incubation with primary antibody (anti-α-SMA, CD68) at 37°C for 1 h. Slides were then washed 3 times with PBS and incubated with secondary antibody for 30 min.

### Confocal microscopy for detection of hepatocyte apoptosis detection and KC and HSC interactions

First, terminal deoxynucleotidyl transferase-mediated dUTP nick-end labeling (TUNEL) assay were performed using a commercially available kit (Chemicon, Internat. Inc.) following the manufacturer’s instructions. then, slides were incubated in 5% BSA for 30 min, and followed by incubation with primary antibody (anti-Heppar) at 37°C for 1 h.washed 3 times with PBS and they were incubated with secondary antibody (Cy3-conjugated affinipure goat anti-mouse) for 30 min.

For double-color labeling of CD68 and α-SMA, liver sections were incubated with monoclonal anti-CD68 for 1 h at room temperature, biotin-conjugated anti-mouse IgG1 (A85-1, BD Pharmingen Inc., San Diego, CA, USA) was then added, and the mixture was incubated for 30 min. The staining reaction was developed with a Cy3-conjugated streptavidin incubation for 30 min and, after washing, the sections were incubated with α-SMA antibody followed by Alexa Fluor 647-labeled goat anti-mouse isotype-specific antibody and then covered with mounting medium.

### Statistical analysis

All results were expressed as mean and standard deviation. Measurement data were analyzed using a one-way analysis of variances (ANOVA, SPSS, Inc., Chicago, IL, USA). Rank data were analyzed with ridit. Groups were compared using ANOVA with Dunnett’s multiple comparison test. Results with *p* < 0.05 were considered to be statistically significant.

## Results

### HQD inhibited HSC activation but did not significantly affect collagen content in DMN livers

**Figure 1:**
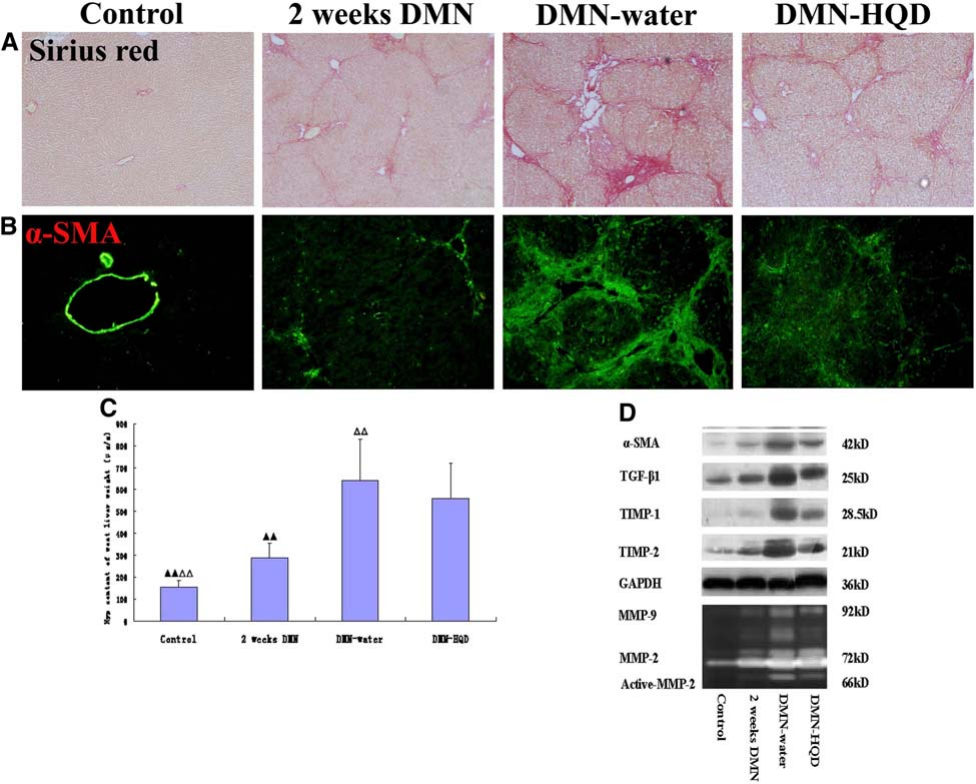
**Effects of Huangqi decoction (HQD) on histological changes (A, Sirius Red staining original magnification × 100; B, α-SMA staining original magnification × 100), C, hydroxyproline content, and D, the expression of pro-fibrotic factors of rat livers in DMN-induced liver fibrosis. **^▴^ p < 0.05, ^▴▴^p < 0.01, vs. DMN-water rats; ^△^p < 0.05, ^△△^p < 0.01 vs. 2 weeks DMN rats. Western blot analysis of total protein extracts was performed by antibodies recognizing α-SMA, TIMP-1, TGF-β1 and GAPDH respectively. Results are mean ± SD.

Observations of Sirius red staining showed that collagen was scarce except around small central venous walls in control liver (Figure 1A). In DMN-treated livers at 2 weeks, collagen was seen to stretch from the portal area to lobular areas, and incomplete septa were also observed. In DMN-water liver, cirrhotic nodule formation was observed and, in comparison, collagen deposition was decreased to a certain extent in DMN-HQD liver. Ridit analysis showed that the DMN-HQD rats were not significantly different from the DMN-water rats (*p* > 0.05). (Table 1).

**Table 1:** Effects of HQD on fibrotic grade and hydroxyproline (Hyp) content in DMN-induced liver fibrosis of rats

**Group**	***n***	**Hyp content ug/g(**$\bar{x}$**±*s)***	**Fibrotic grade**					**Confidence interval**
			**0**	**I**	**II**	**III**	**IV**	
Control	10	179.36 ± 31.29^▴▴△△^	10	0	0	0	0	(-0.110,0.228)^▴▴△△^
2 weeks DMN	6	289.06 ± 66.00^▴▴^	0	0	4	2	0	(0.051,0.486)^▴▴^
DMN-water	12	641.71 ± 187.81^△△^	0	0	0	3	9	(0.586,0.894)^△△^
DMN-HQD	12	559.04 ± 163.02	0	0	0	8	4	(0.580,0.734)

grade 0: normal; grade 1: very slight; grade 2: slight; grade 3: moderate; grade 4: severe. Data are expressed as numbers of animals with each fibrotic grade. P < 0.05 P < 0.01 (vs DMN- water) P < 0.05 P < 0.01 (vs 2 weeks DMN).

The inhibitory effect of HQD treatment against fibrosis progression in a quantitative manner was evaluated by measuring liver Hyp content. HQD was not found to decrease liver Hyp content significantly *(p* > 0.05, Figure 1C).

As sustained deposition of extracellular matrix resultes mainly from HSC activation, a correlation between accumulated collagen and activated HSCs was studied by detecting a-SMA, a marker of activated HSC, immunohistochemically in liver sections, as described above. Vascular smooth muscle cells were positive for α-SMA in control liver (Figure 1B). α-SMA^+^ HSCs were detected in periportal fibrotic bands in 2-week DMN liver, with the number of α-SMA^+^ HSCs increasing gradually to a peak at 4-week DMN-water cirrhotic liver. In contrast, a marked reduction of α-SMA^+^ HSCs was observed in DMN-HQD liver, compared with DMN-water liver.

Altered expression of α-SMA in DMN-treated liver samples was also detected by western blot analyses (Figure 1D). The expression of transforming growth factor (TGF)-β_1_, tissue inhibitor of metalloproteinases (TIMP)-1, and TIMP-2 increased gradually following DMN treatment and these trends were almost identical with that of α-SMA. Compared to DMN-water liver, HQD administration resulted in marked reductions in TGF-β_1_ and TIMP-1/2, and control rats expressed little active matrix metalloproteinases (MMP)-2/9 (Figure 1D). Following DMN treatment, activities of these enzymes were increased significantly and, in addition, less intense bands of 66 kD were present in zymograms of liver protein from DMN-water treatment, which corresponded to partially or completely activated forms of MMP-2. In contrast, treatment with HQD showed reductions in MMP-2/9 activity.

Taken together, these results confirmed that DMN administration caused HSC activation and increased production and accumulation of extracellular matrix, which might have facilitated or resulted in liver fibrosis and cirrhosis in DMN-treated rats. HQD remarkably decreased α-SMA^+^ HSCs, whereas HQD did not significantly reduce collagen content.

### HQD was reduced on hepatocyte apoptosis and pro-apoptotic factors

The fact that, in rat liver, HQD significantly inhibited HSC activation but did not significantly decrease collagen was puzzling. As liver fibrosis is a complex process resulting from hepatocyte apoptosis, inflammation, and HSC activation, in liver fibrosis development, hepatocyte apoptosis and KC and HSC interactions lead to fibrosis. Thus, some important factors involved in these processes were detected here.

**Figure 2:**
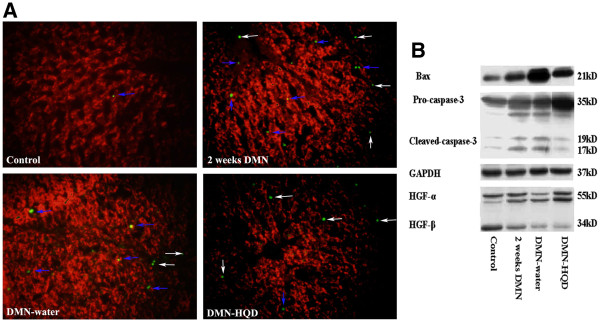
**Effects of HQD on hepatocyte apoptosis (A), caspase-3 and HGF expression (B) in DMN-induced liver fibrosis in rats. **Hepatic cryosections were stained with TUNEL (green) and hepatocytes paraffin antigen (Heppar, red). TUNEL (green) located in or near Heppar (red) means hepatocyte apoptosis. The arrows with blue color indicate apoptotic hepatocytes, the arrows with white color indicate non-hepatocyte apoptosis. (Original magnification × 400).

DMN is known to induce hepatocyte apoptosis [18,19]. Here, hepatocyte apoptosis was studied using confocal microscopy characterized by hepatocyte paraffin antigen (Heppar, a hepatocyte marker) with TUNEL. The results showed that hepatocyte apoptosis was rarely observed in control liver (Figure 2A), but there were clear apoptotic hepatocytes detected in 2-week DMN liver. In 2- or 4-week DMN liver, there were a few hepatocyte apoptoses as well as some other apoptotic cells and , HQD inhibited hepatocyte apoptosis.

The expression of Bax and caspase-3 was found to be significantly increased following 2 weeks of DMN treatment (Figure 2B) and decreased in DMN-water liver. HQD administration resulted in significant reductions in cleaved-caspase-3 and Bax expression, compared with DMN-water liver. HGF-α expression decreased gradually following DMN administration (Figure 2B), and compared with DMN-water liver, HQD remarkably increased HGF-α expression.

### Effects of HQD on proinflammatory factors such as CD68, TNF-α, IL-1β, and MIP-1α

Liver inflammation can be associated with KC activation and trigger migration of “professional” phagocytes into hepatic cords where these macrophages stimulate fibrosis by secreting proinflammatory cytokines. To elucidate the roles of KCs in DMN-induced liver fibrosis, a specific KC marker, CD68, has been used to monitor KC activation [20].

**Figure 3:**
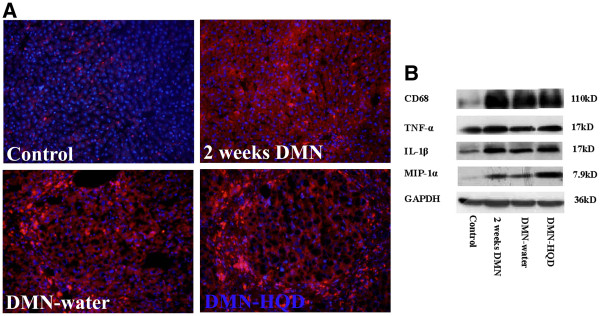
**Effects of HQD on CD68 using immunofluorescence staining (A) and the expression of pro-inflammatory factors (B) using western blot analysis in DMN-induced liver fibrosis. **Western blot analysis of total protein extracts was performed by antibodies recognizing CD68, TNF-α, MIP-1, IL-1, and GAPDH respectively. CD68, red; DAPI, blue; (original magnification × 200).

Here, CD68^+^ KCs were observed in hepatic sinusoid while they were extremely low in control liver (Figure 3A). After 2 weeks of DMN administration, strongly stained CD68^+^ KCs not only appeared in hepatic sinusoid, but were also found in the portal area. CD68^+^ KCs were mainly located in the fibrotic band and portal area in DMN-water liver. HQD was found to increase CD68^+^ KCs expression in fibrotic septa, results similar to those were obtained from western blot analysis (Figure 3B).

Compared with control liver, CD68 expression increased dramatically with 2-week DMN liver (Figure 3B). CD68 decreased significantly in 4-week DMN-water liver compared with 2–week DMN liver, and significantly increased in DMN-HQD compared with 4-week DMN-water liver (Figure 3B). The expression trends of TNF-α, IL-1β and MIP-1α were almost identical with that of CD68, which indicated that, here, CD68^+^ KCs were the major source of hepatic proinflammatory responses**.**

### Effect of HQD on KC and HSC interaction

**Figure 4:**
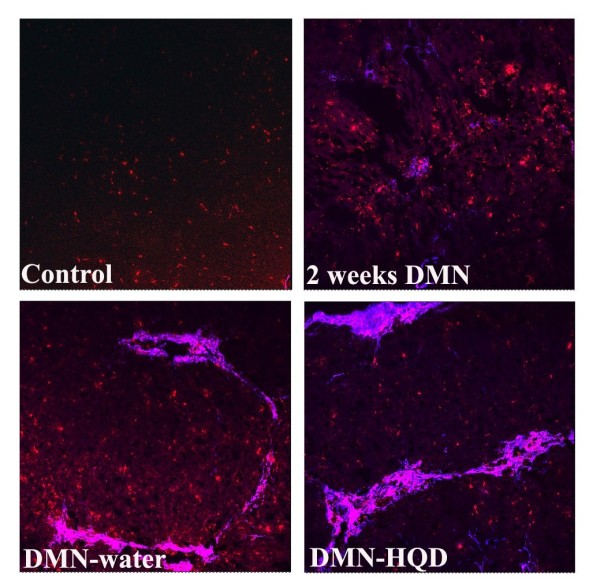
**Effects of HQD on interaction of KCs and HSCs in DMN-induced liver fibrosis in rats. **Hepatic cryosections were stained with α-SMA (blue) and CD68 (red). The pink color means the colocalization of (CD68) KCs and (α-SMA) HSCs. (Original magnification × 200).

The pathophysiological involvement of KCs in DMN-induced liver fibrosis was elucidated by observation of colocalized CD68 and α-SMA using confocal microscopy. From double-color immunofluorescence staining, CD68^+^ KCs were observed neighboring or directly adhered to the α-SMA^+^ HSCs in the sinusoid or portal area in 2-week DMN liver, while both cell types were not observed in control liver (Figure 4). Similar results were also observed in 4-week DMN-water liver. HQD was found to reduce α-SMA expression but also to increase CD68 expression. In addition, widespread KC to HSC adhesion was observed in thick fibrotic bands in DMN-HQD liver. These results collectively demonstrated that KCs played a key role in inflammation and fibrosis from DMN-induced liver injury.

## Discussion

The present study demonstrated that HQD ameliorated liver fibrosis to some extent and in a comprehensive manner. HQD exerted a beneficial anti-apoptotic influence and significantly inhibited fibrotic-related factors remarkably. However, it increased the expression CD68 and proinflammatory cytokines in the presence of DMN-induced liver fibrosis. These results indicated that KC activation and inflammation played a key role in the process of liver fibrosis to cirrhosis development.

DMN-induced liver fibrosis in rats is a reproducible model for studying the pathogenesis of liver fibrosis and cirrhosis [21,22]. Hepatocyte apoptosis is a cardinal feature of many liver diseases and is thought to play a role in initiating and maintaining HSC activation. Apoptosis of parenchymal cells is no longer viewed a silent consequence of liver injury, but rather as an important inflammatory stimulus that activates stellate cells, which display a surprising capacity to phagocytize apoptotic bodies. Thus, efforts to block hepatocyte apoptosis therapeutically are being developed as a potential antifibrotic strategy.

HGF is a pluripotent growth factor displaying a remarkable ability to promote tissue repair and organ regeneration after injury. and indeed appears to ameliorate fibrosis and cirrhosis in animal models [23-26]. Three different mechanisms have been proposed for HGF's antifibrotic effects. One is the prevention hepatocyte apoptosis and stimulation of hepatocyte mitosis in DMN-induced liver fibrosis, resulting in the survival of rats with an otherwise lethal illness. the second is an increase in hepatic collagenase activity that promotes degradation of the extracellular matrix (ECM) components. The third pathway is the reduced expression of of mRNA for procollagens and TGF-β1. the latter being a crucial factor in liver fibrosis and a potent inhibitor of hepatocyte growth. It has been demonstrated that HGF antagonizes TGF-β1 directly, improving liver regeneration by inhibiting TGF-β1 expression [23]. The specific mechanism responsible for improved liver functional capacity with HGF remains unclear. but recently observations have suggested that HGF is a potent anti-inflammatory cytokine that inhibits renal inflammation by disrupting nuclear factor (NF)-κB signaling and can be a promising therapeutic agent for progressive renal diseases. The present data showed that HGF decreased gradually during the course of DMN-induced liver fibrosis. In contrast, HGF-α expression in DMN-HQD liver was increased significantly compared to DMN-water liver.

Liver fibrosis is traditionally viewed as a progressive pathological process involving multiple cellular and molecular events that ultimately lead to deposition of excess matrix proteins in the extracellular space [27]. Studies with both animal and human fibrogenesis have demonstrated a positive correlation between the degree of fibrosis and HSC activation in damaged liver [28]. Activated HSCs are characterized by a high rate of proliferation, the expression of fibrotic cell markers such as α-SMA, and the production of ECM. In the present study, western blotting and immunofluorescence staining of α-SMA showed that HSCs were activated in DMN-induced liver fibrosis and that HQD significantly suppressed HSC activation significantly.

From the current therapeutic point of view, suppression of hepatocyte apoptosis and profibrogenic factors and increases in HGF expression are critical in the prevention of fibrosis onset and progression. As here HQD increased HGF-α expression, decreased cleaved-caspase-3 expression, and suppressed α-SMA expression, HQD exhibited both anti-apoptotic and fibrinolytic potential, as described above. Thus, HQD appears to be an ideal therapeutic agent for liver fibrosis treatment. However, the hydroxyproline assays and histological evaluations demonstrated that HQD had certain but not notable effects in DMN-induced liver fibrosis. Taken together these results indicated there were some more important pathological factors involved in DMN-induced liver fibrosis.

KCs synthesize a variety of mediators, such as proinflammatory cytokines and reactive oxygen species, and fulfill a crucial role in the liver immune response [29-31]. In the chronic inflammatory milieu, KCs interact with other cell types, including fibroblasts that transdifferentiate into matrix-secreting myofibroblasts, with resultant scar-tissue formation and disruption. Accumulating evidence has suggested that KCs play a key role in regulating HSC activation and function, and thus, a thorough our understanding of the complex interplay between chronic inflammation and progressive fibrosis is a critical step toward the rational design of new treatments; several studies have evaluated the role of conditioned medium from KCs in stimulating HSCs [32,33]. However, to date there has been limited available information regarding the interaction of KCs and HSCs *in vivo*. Meanwhile cell culture (*in vitro*) does not properly reflect the complexity of microenvironment *in vivo*, and interaction or microenvironment *in vivo* should be considered the gold standard for the study of HSC and KC biology. The results from double-color confocal evaluation of CD68 and α-SMA, results showed that KCs were located adjacent to or directly interacting with α-SMA-positive HSCs in the DMN-water liver, indicating that KC activated HSC in fibrotic septa. HQD reduced α-SMA expression but increased CD68 expression, such that HQD did not inhibit the interaction of KCs and HSCs. From these results, it was concluded that KCs or inflammation played a vital role in regulating fibrogenesis in DMN-induced liver fibrosis.

In summary, the present observations demonstrated that HQD suppressed expression of HSC activation and hepatocyte apoptosis, while also increasing HGF-α expression. However, it did not suppress the expression of proinflammatory factors, such ad CD68 and TNF-α. The results clearly indicated that KCs played a vital role in DMN-induced liver fibrosis and, collectively, augment the understanding of the mechanism of hepatic fibrosis. In addition, they might provide potential for the development of therapeutic manipulations for modulating fibrosis through the suppression of KC activation and proinflammatory cytokines.

## Conclusion

HQD has favorable ameliorating effects against liver fibrosis; its mechanism of actions was associated with protection from hepatocyte apoptosis and promotion of CD 68 expression in the development of liver fibrosis to cirrhosis development.

## Abbreviations

α-SMA: smooth muscle actin alpha; DMN: dimethylnitrosamine; GAPDH: glyceraldehyde-3-phosphate dehydrogenase; Heppar: hepatocyte paraffin antigen; HGF: hepatocyte growth factor; HSCs: hepatic stellate cells; KCs: Kupffer cells; MMPs: matrix metalloproteinase; TGF-β: transforming growth factor beta; TIMPs: tissue inhibitor of metalloproteinases; TNF-α: tumor necrosis factor alpha; TUNEL: terminal deoxynucleotidyl transferase-mediated dUTP nick-end labelling; HQD: Huangqi decoction.

## Competing interests

The authors declare that they have no competing interests.

## Authors' contributions

CL and PL conceived the study design. CL, YM, LZ,XH, GW and GC performed the data analysis. CL drafted and revised the manuscript. All authors confirm that the content of this paper has not been published elsewhere and does not overlap or duplicate their published work. All authors have read and approved the final manuscript.

## Pre-publication history

The pre-publication history for this paper can be accessed here:

http://www.biomedcentral.com/1472-6882/12/51/prepub
